# Assessment of an iPad-based sperm motility analyzer for determination of canine sperm motility

**DOI:** 10.1093/tas/txab066

**Published:** 2021-04-10

**Authors:** Evelyn Bulkeley, Christine Collins, Azarene Foutouhi, Kris Gonzales, Heather Power, Stuart Meyers

**Affiliations:** 1 Department of Anatomy, Physiology, and Cell Biology, School of Veterinary Medicine, University of California, Davis, CA, USA; 2 Veterinary Services, Guide Dogs for the Blind, San Rafael, CA, USA

**Keywords:** canine, CASA, iSperm, motility, sperm

## Abstract

The objective of this study was to evaluate the repeatability and accuracy of canine sperm motility (total and progressive) assessment with a tablet-based Canine iSperm instrument compared to computer-assisted sperm analysis (CASA). The experiment used fresh and frozen/thawed canine semen samples for comparisons of semen analysis parameters (concentration, total motility, and progressive motility) between a CASA system, iSperm, and NucleoCounter SP-100 (concentration) instruments. Spearman’s Rho correlational analysis was used to identify significant associations between motility assessment methods. Significant positive correlations were found between CASA assessment and iSperm for both progressive and total motility measurements. We also determined the coefficient of variation (CV) for repeatability of sample analysis for iSperm and CASA for fresh sperm, wherein each sample was assessed 10 times on both devices. For fresh and frozen-thawed samples, concentration assessment by iSperm showed high variability (CV= 19.9 ± 1.5%). For iSperm assessment of total and progressive motility, the CVs were 6.3 ± 0.5% and 10.7 ± 0.8%, respectively. The results indicate that the iSperm application offers an accurate and alternative measurement of motility to traditional CASA analysis, though caution should be taken when assessing concentration due to the high CV observed in this study.

## INTRODUCTION

Microscopic assessment of sperm in motion has been used as a clinical and research tool for human and animal reproductive health over the past 340 years. Antoni van Leewenhoek reported microscopic sperm morphology in 1678 for human and dog sperm (Ruestow, 1983) using an early single-lens microscope (Amann and Katz, 2004; Amann and Waberski, 2014). In the past 40 years, the advancement of computer-assisted sperm analysis (CASA) has highlighted the ability to objectively assess large populations of motile and immotile sperm and has been summarized in considerable detail (Amann and Waberski, 2014).

Visual assessments of canine sperm motility are subject to observer bias and for this reason, the use of automated or computerized systems has been growing for wider clinical usage. Automated or computerized assessment of sperm motility, a sperm physiological parameter that is necessary for fertilization in vivo, has grown as a standard of practice for human fertility clinics as well as livestock breeding. The animal agriculture industry has recently taken advantage of new low-cost microprocessors and software for on-farm use. Automated sperm motility analysis has not been widely available to canine breeding programs which are largely performed by veterinary and lay breeding personnel and some specialty veterinary practices. CASA has been successfully employed for accurate motility assessment in dogs (Ellington et al., 1993; Walker et al., 1982); however, due to high costs and bench-space limitations, few small animal veterinary practitioners have access to CASA systems (Root Kustritz, 2007).

On-site semen evaluation has pushed technology developers to produce affordable, objective, and portable systems that can use a tablet-based camera with warming lenses that support real-time animal-side sperm analysis. The objective of this study was to assess the accuracy and reliability of a new and inexpensive tablet-based CASA system for sperm motility evaluation. We evaluated the iSperm instrument for repeatability and accuracy in the assessment of canine sperm motility. Additionally, we assessed the repeatability of sperm concentration assessment by the iSperm.

## METHODS

### Chemicals and Reagents

iSperm sampling chips were obtained from GenePro (Fitchburg, WI). NucleoCounter SP-100 cassettes and reagents were purchased from ChemoMetec (Allerød, Denmark). All other chemicals were obtained from Sigma Chemical Co. (St. Louis, MO) unless otherwise stated. A modified Tyrode’s medium (TALP) was used for all experiments (Parrish, 2014). This media consisted of 1 mg/mL PVA, 81 mmol/L NaCl, 2.8 mmol/L KCl, 0.2645 mmol/L KH_2_PO_4_, 40 mmol/L HEPES sodium salt, 2 mmol/L NaHCO_3_, 2 mmol/L CaCl_2_ (0.1 M solution, Ricca), and 0.4 mmol/L MgCl_2_ (1 M solution). Media metabolites consisted of 5 mmol/L D-glucose, 1 mmol/L sodium pyruvate, and 0.1862% (v/v; 21.6 mmol/L) DL-Lactic acid syrup and pH was adjusted to 7.4 ± 0.02 and osmolality of 300 ± 10 mOsm/kg.

### Animals

Fresh semen was collected by manual collection at the Guide Dogs for the Blind (GDB; San Rafael, CA) from dogs (*N* = 5) owned by GDB that resided with a guardian owner in the San Francisco metropolitan area. They were under the medical care of veterinarians and staff at GDB during visits to the GDB campus and all animals were current in vaccinations. The dogs were, fed similar diets, and in good overall health per the annual veterinary evaluation at GDB. For cryopreserved semen, ejaculates were collected at the University of California, Davis Veterinary Medicine Teaching Hospital (VMTH) under IACUC-approved guidelines on an out-patient basis from dogs (*N* = 4) with signed owner consent from Golden Retriever (*N* = 1), Newfoundland (*N* = 1), and Labrador Retriever dogs (*N* = 2) by digital manipulation at UC Davis Veterinary Medicine Teaching Hospital under University of California IACUC-approved guidelines.

### Semen Collection and Processing

***Fresh semen.*** Fresh semen was collected from Labrador (*N* = 3) and Golden Retrievers (*N* = 2) by a veterinarian using a standard manual method and teaser female. Semen was collected into sterile plastic funnels attached to 15 mL conical tubes after the dog achieved erection during the mount. Semen was processed immediately for ejaculate volume and concentration and subjective initial motility evaluation on a phase-contrast microscope at 400× magnification was performed at GDB. The samples were then diluted 1:2 (semen to extender ratio) using TALP and transported at ambient temperature by car for the 1 hour trip to the lab at UC Davis. Upon arrival in the lab, diluted semen samples were assessed for initial concentration using a Nucleocounter SP-100 by following the manufacturer’s guidelines. A significant decline in sperm motility was not observed following transport in any samples. The semen samples were then diluted and concentrations were re-evaluated by a Nucleocounter SP-100 to ensure concentrations were between 30–60 million sperm/mL, the manufacturer recommended concentration range for canine iSperm assessment. The diluted samples were then placed in an incubator at 37 °C on their sides to prevent sperm compaction at the bottom of the tube and only removed from the incubator to retrieve a sample to be used for iSperm and CASA.

***Cryopreserved semen.*** Ejaculates were manually collected on an out-patient basis at the UC Davis VMTH from Golden Retriever (*N* = 1), Newfoundland (*N* = 1), and Labrador Retriever dogs (*N* = 2). Ejaculate volume was recorded and concentration and viability were quantified using a Nucleocounter SP-100 (ChemoMetec, Allerod, Denmark). To estimate sperm motility for freezing, Initial sperm motility parameters were quantified using CASA (SpermVision SAR system, Minitube USA Inc., Verona, WI, USA). Seminal plasma was separated from sperm cells using Semen Separating Solution (Zoetis Inc, Parsippany-Troy Hills, NJ, USA) in preparation for freezing. Sperm was diluted 2:1 using Canine Freeze Buffer (Zoetis), and adjusted to a final concentration of 150 × 10^6^ sperm/mL using the addition of Zoetis Dilution Buffer (Zoetis Inc.). Samples were loaded into 0.50 mL straws (Agtech Inc, Manhattan, KS, USA) and carefully sealed with polyvinyl alcohol powder. Cryopreservation was performed using a Planar Kryo 10 Series III controlled rate freezer (Planar Limited, Middlesex, United Kingdom). Straws were cooled to 10 °C beginning at room temperature 24 °C, using the cooling rate of −0.3 °C/min which was completed in 20 minutes. Straws were cooled over the next 60 minutes at a rate of −0.2 °C/ min until reaching 4 °C, then −10 °C/ min until reaching −15 °C, then −17 °C/ min until reaching −110 °C. Straws were removed from the Planar freezer and immediately plunged in liquid nitrogen (−196 °C). At thawing, the frozen semen straws were removed individually from liquid nitrogen storage tanks and placed into a warm water bath at 37 °C for 30 seconds. After 30 seconds, the semen straw was cut on both ends and the contents decanted into a tube. These samples were then checked for their concentration and diluted as described above for fresh samples.

### Sperm Analysis: Comparison between iSperm and SpermVision SAR

The iSperm software (Aidmics Biotechnology Co., Ltd, Taipei City 10647, Taiwan) and instrumentation included an iPad Mini (Apple Inc., Cupertino, CA, USA) plus the proprietary microscope camera (Aidmics) which were set up according to the guidelines of the iSperm instruction manual. When conducting all of the trials, a sample of 7.5 μL was collected using a micropipette and placed onto the surface of the base chip, then flipped over into the cover chip and pressed for one to two seconds on the countertop, as per the instruction manual. The base chip plus cover chip were then screwed into the microscope attached to the iPad Mini camera in order to be analyzed. Each semen sample (fresh ejaculate or frozen-thawed straw) was assessed using three separate iSperm sample chips (e.g. in triplicate), and each sample chip was analyzed ten times consecutively in the iSperm application to allow for later calculation of the coefficient of variation (CV). The same orientation was used throughout the process of measuring the sample ten times in a row with the “analyze” button being pushed ten times consecutively, providing data acquisition for ten observations. There were no other changes to the sample chip until analysis was completed and a new chip was sampled. The amount of time taken to analyze one sample chip ten times ranged from three to five minutes. The parameters quantified and collected in the iSperm application included total motility (%), progressive motility (%), and concentration (M/mL).

For CASA, 3.5 μL semen samples were loaded into each well of four-chambered Leja slides (Leja Products BV, Luzernestraat 10, The Netherlands). The slides were then placed on a warming plate (37 °C) for five minutes. The slide was then placed on the warmed (37 °C) stage of a Zeiss AxioLab A1 phase-contrast microscope, connected to a PC laptop equipped with SpermVision SAR software (Minitube USA). Each sample was loaded into three separate chambers for motility assessment by SpermVision SAR (eg. in triplicate). Per manufacturer recommendations, the CASA system motility analysis was performed for each slide chamber by recording motility parameters of seven different microscopic fields and averaging the seven for endpoint analysis of the sample. Because the iSperm manual provided limited information regarding motility parameter settings and due to inherent differences between the iSperm and SpermVision software, it was not possible to exactly match the motility settings between the two instruments for comparison. However, to allow the best possible direct comparison between the two modalities, all possible adjustments were made to match motility settings for SpermVision analysis to the defined iSperm settings. This was accomplished using the SpermVision manufacturer-recommended motility settings for canine sperm analysis as a base template. CASA assessment using these modified settings will be referred to as “CASA_iSperm.” For additional comparison, all samples were also analyzed using the unmodified manufacturer-recommended settings for canine motility assessment, which will be referred to as “CASA_canine.” In summary, two separate CASA analyses were performed on each sample, one using the manufacturer-recommended canine SpermVision settings (“CASA_canine”), and another analysis using SpermVision settings manually adjusted to best match the settings programmed by iSperm (“CASA_iSperm”). The parameters of interest that were collected and analyzed by the CASA included total motility (TM, %) and progressive motility (PM, %).

### Data Management and Statistical Analysis

Data from each assessment method (iSperm, CASA_canine, and CASA_iSperm) was exported to Microsoft Excel. The CV, or relative standard deviation (rsd), was calculated [(sd/mean)*100] for each of the iSperm sample chips for TM, PM, and concentration data. The ten consecutive measurements recorded for each parameter were averaged for all additional analyses. Exploratory and statistical analyses were performed using JMP statistical software (version 14.0.0; SAS Institute Inc., Cary, NC, USA). Data distributions were checked for normality with the Shapiro-Wilk test. Nonparametric Spearman’s Rho (*r*_*s*_) correlational analysis was then performed to identify significant associations between assessment methods. Comparisons between TM and PM measurements between assessment methods were carried the Wilcoxon method.

## RESULTS

For fresh and frozen-thawed samples, concentration assessment by iSperm showed high variability (CV = 19.9 ± 1.5%). The very low sperm motility of the post-thaw samples resulted in a high variation during consecutive motility assessments for CV calculation (Table 1). Consequently, only fresh samples (*N* = 15) were used to evaluate the CV of motility measurements. For iSperm assessment of total and progressive motility, the CVs were 6.3 ± 0.5% and 10.7 ± 0.8%, respectively.

**Table 1.** CV determinations for iSperm concentration and motility in fresh semen (*N* = 15, *P* < 0.05)

	Concentration		Total motility		Progressive motility	
	CV	SEM	CV	SEM	CV	SEM
Fresh semen	22.97	1.50	6.13	0.46	10.68	0.80

**Figure 1.**
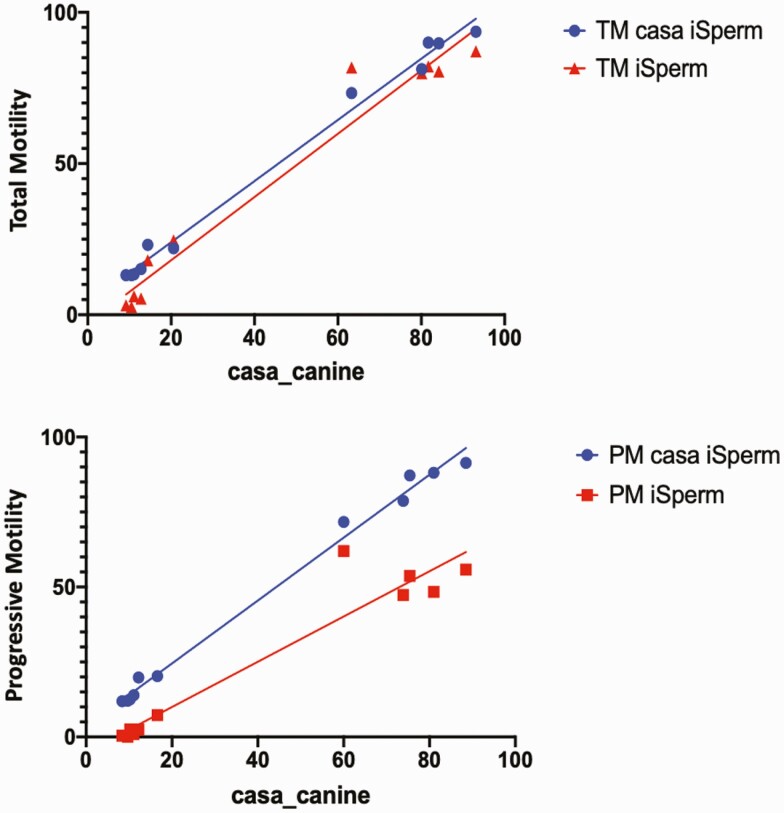
Comparison of assessment methods for (A) TM and (B) PM measurements.

Spearman’s Rho correlational analysis was used to identify significant associations between motility assessment methods because the data were not normally distributed. Strong significant positive correlations were found between all assessment methods for both TM and PM measurements. Specifically, TM measurements were strongly correlated between the iSperm and both the CASA_iSperm (*r*_*s*_ = 0.95, *P* < 0.0001) and the CASA_canine (*r*_*s*_ = 0.93, *P* < 0.0001), as well as between the CASA_iSperm and CASA_canine (*r*_*s*_ = 0.97, *P* < 0.0001) (Fig. 1A). Strong positive correlations were also identified for PM measurements between the iSperm and both CASA_canine (*r*_*s*_ = 0.87, *P* = 0.0004) and CASA_iSperm (*r*_*s*_ = 0.87, *P* = 0.0004), in addition to between CASA_iSperm and CASA_canine (*r*_*s*_ = 1.00, *P* < 0.0001) (Fig. 1B).

**Figure 2.**
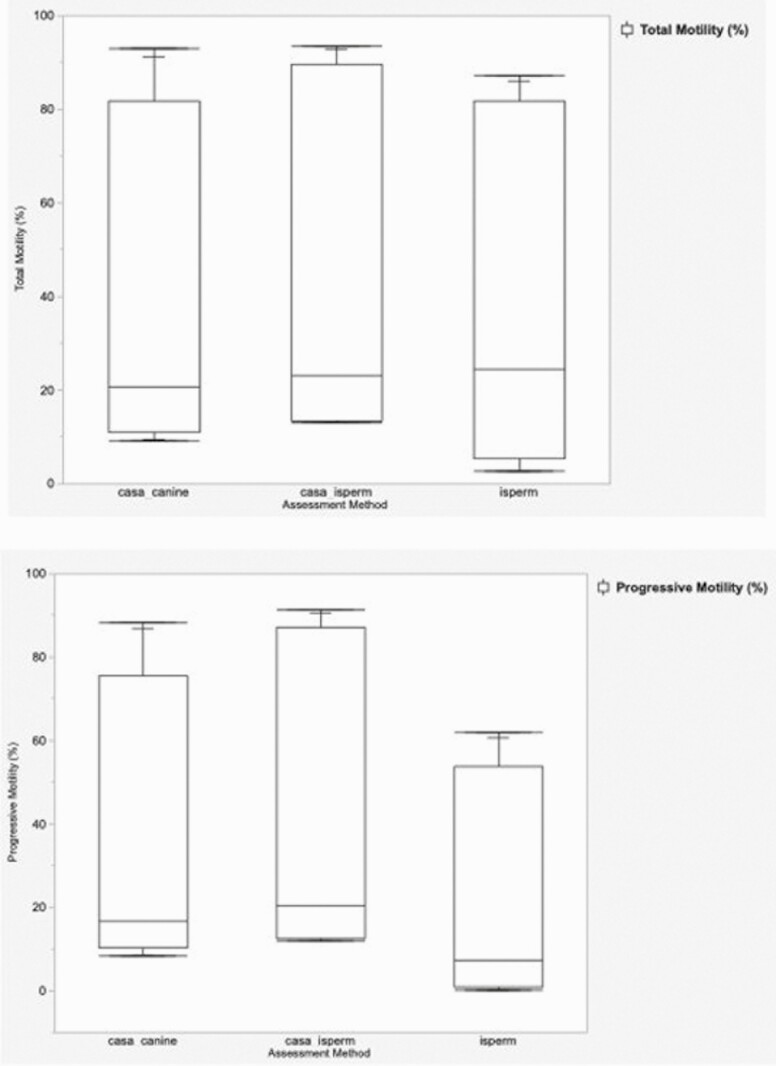
Box plots demonstrating canine sperm total and progressive motility measurements by assessment method. (A) Total motility (B) Progressive motility.

Comparisons between TM values between assessment method revealed no significant differences between the iSperm and CASA_canine (*Z* = −0.39, *P* = 0.69) or CASA_iSperm (*Z* = −0.79, *P* = 0.43). No significant differences were identified between CASA_iSperm and CASA_canine (*Z* = 0.92, *P* = 0.36) (Fig. 2A). Comparisons between PM measurements revealed a slight, although significant difference between iSperm and CASA_iSperm (*Z* = −1.97, *P* = 0.05), but no significant difference between iSperm and CASA_canine (*Z* = −1.90, *P* = 0.06). No significant differences were observed in PM measurements between CASA_iSperm and CASA_canine (*Z* = 1.05, *P* = 0.29) (Fig. 2B).

## DISCUSSION

The iSperm Canine software was created to provide a relatively inexpensive and portable device that can be used for semen analysis as an animal-side test in clinical and field conditions, such as veterinary hospitals, dog shows and competitive sporting events where semen evaluation is commercially performed. The iSperm was designed to measure sperm concentration and motility parameters (including total and progressive motility) abnormality, VCL, VAP, VSL, STR, and LIN of a sample, which can then be used to calculate an extended semen dose for breeding. Our goal was to assess the iSperm Canine application’s repeatability by assessing CV values for concentration, total and progressive motility measurements, as well as to validate and assess the accuracy of motility assessment by the iSperm Canine application through comparison to motility assessment by CASA, the “gold standard” of semen motility assessment.

The commercial CASA instrument used for validation, Sperm Vision SAR, has been widely used to assess sperm motility and morphology through digitized video recordings of sperm. This instrument computes their motion tracks which can then be analyzed and then standardized (Spiropoulos, 2001). The iSperm application utilizes a similar process to calculate sperm concentration and motility by using video recordings through the iPad Mini to calculate the trajectory and concentration of the sperm in the sample but uses different and proprietary algorithms and software for the process. Due to inherent differences in software and minimal details available regarding the iSperm motility parameter settings (threshold values, etc.), it was not possible to completely match the CASA parameter settings between the two systems (iSperm and CASA). For this reason, we compared motility assessment with the iSperm Canine application to CASA assessment using both the Sperm Vision manufacturer-recommended canine settings (CASA_Canine), and a version of those settings modified to best match the preprogrammed settings of the iSperm Canine application (CASA_iSperm).

Correlational analysis with Spearman’s Rho revealed significant strong positive correlations in both TM and PM measurements between all three assessment methods (iSperm Canine application, CASA_iSperm, and CASA_canine). For TM assessment, the correlation was slightly stronger between the iSperm Canine application and CASA_iSperm than with CASA_canine, but the correlation for PM assessment was comparable between the iSperm Canine application and both the CASA_iSperm and CASA_canine. Comparison of TM and PM values between analysis methods by the Wilcoxon method overall did not reveal significant differences, with the exception of a slight, although significant, the difference in PM values between the iSperm and CASA_iSperm. Overall, these results demonstrate the accuracy of the iSperm application for measuring the total motility in canine semen. These results also suggest reasonable accuracy of the iSperm for PM assessment. Although a very slight significant difference was detected between PM values with the CASA_iSperm, no significant differences were detected in PM values between the iSperm Canine application and the CASA_canine. This, in combination with the high positive correlation seen in PM measurements between the iSperm canine application and both settings used for CASA assessment, leads us to conclude that iSperm Canine application also demonstrates reasonably accurate progressive motility assessment.

Dini and colleagues (2019) determined the iSperm for horses had different results on the ability of iSperm for the concentration of a semen sample (Dini et al., 2019). They reported a higher correlation between the iSperm and the Nucleocounter SP-100 and less than 10% difference when measuring semen samples between 20 and 100 M/ml. In contrast. our results did not identify a high correlation between the two methods of estimating sperm concentration. However, similar to our findings, Dini et al. (2019) showed high correlations between the iSperm and Nucleocounter SP-100 when measuring semen concentrations in samples on the lower side of the iSperm indicated range. Just as our results identified a strong correlation between CASA and iSperm for the measurement of progressive motility, Dini et al. (2019) similarly reported a strong positive correlation between iSperm and Androvision. This demonstrates the utility and accuracy of the iSperm for the portable measurement of progressive motility for different species.

Although the motility data from this study did not follow a normal distribution, that was expected, as both high-quality fresh semen and poor-quality frozen semen were evaluated in this study. By analyzing semen with low motility and semen with excellent motility, we were able to observe the maximum ranges of the iSperm and determine its accuracy for these points. We analyzed very high-quality semen that had high total and progressive motilities from dogs that have been selected as having outstanding fertility and semen quality in the GDB Breeding Center. Our other semen samples came from out-patient dogs, which had not been bred and selected for high fertility and often had poor fresh semen quality. The initial deficits in semen quality were compounded by the process of cryopreservation, which is known to result in as much as a 50% loss of motility in a single freeze-thaw cycle (Nöthling and Shuttleworth, 2005). Thus, the sperm motilities observed in our experiment were very low in frozen-thawed semen, resulting in the distribution veering from normality.

In this study, we assessed the repeatability of sperm concentration and motility assessment with the iSperm Canine. CVs and this for motility measurements were only assessed for fresh ejaculates due to the high variability inherent to the frozen-thawed samples with very low motility. Total and progressive motility assessment of fresh semen by the iSperm canine application revealed relatively low variability, with CVs of 6.3% and 10.7%, respectively. Pooled observations of fresh and frozen-thawed samples revealed high variability in concentration assessment by iSperm Canine.

Moraes et al. (2019) concluded that the iSperm Equine application was a valid means for concentration, concentration, total motility, and progressive motility assessment of equine semen. This study found that iSperm Equine assessment of semen concentration did not significantly differ from Nucleocounter or hemocytometer assessment. However, the authors of this study did not investigate the CV for concentration values generated by the iSperm Equine application. In agreement with our findings, Moraes et al. (2019) found that the iSperm Equine application provided accurate TM and PM motility assessment when compared to a CASA system(Moraes et al., 2019). In agreement with the existing literature, our result indicate that the iSperm could be a very useful and affordable tool for onsite analysis of canine semen, though we recommend repeated measures for concentration assessment due to the high CV we observed for this parameter.

One last technical observation worth noting is that although the iSperm is efficient to use when the specimen on the sample chip comes out clean for the sample collector, the methodology of placing a semen sample with a micropipette on a base chip and pressing it into the cover chip accounts for many technical difficulties. For every “good” sample chip there were about three “bad” sample chips that had bubbles in the sample from pressing the base chip into the cover chip. These bubbles made the sample unusable and were discarded. Thus, many sample chips were wasted from the bubbles created by the design of the sample chips. However, with improvements in the chip design, so that bubble formation could be decreased, the iSperm would become more efficient and “field-ready.” Certainly, with more technical experience loading chips, technicians can minimize chip wastage.

## CONCLUSIONS

The iPad-based iSperm Canine application and apparatus is an innovative instrument that can be beneficial for some canine breeding programs. The total motility and progressive motility for fresh and frozen-thawed samples were found to be accurate when compared to a standard CASA system. Concentration measurements, when compared to the Nucleocounter SP-100 were not well correlated. The iSperm demonstrated some inefficiencies regarding the setup of analyzing a sample (i.e. the formation of bubbles in sample chips). Not only does the iSperm provide accurate measures of progressive and total motility that reliably match that of the CASA system, but also supplies the capture of sample images, allowing the user to conduct more objective measures of canine breeding potential in an inexpensive and efficient manner.
